# The role of the home environment in neurocognitive development of children living in extreme poverty and with frequent illnesses: a cross-sectional study

**DOI:** 10.12688/wellcomeopenres.14702.1

**Published:** 2018-12-03

**Authors:** Margaret Nampijja, Robert Kizindo, Barbara Apule, Swaib Lule, Lawrence Muhangi, Andrew Titman, Alison Elliott, Katie Alcock, Charlie Lewis

**Affiliations:** 1 Coinfections Programme, MRC/UVRI and LSHTM Uganda Research Unit, Plot 51-59 Nakiwogo Road Entebbe. P.O. Box 49, Entebbe, Uganda; 2 Department of Psychology, Lancaster University, Fylde College LA1 4YF, Lancaster, LA1 4YF, UK

**Keywords:** home environment, poverty, health status, child, cognitive function

## Abstract

**Background:** The home environment is reported to contribute significantly to children’s developing cognitive skills. However, it is not yet evident whether this role prevails in the context of extreme poverty and frequent ill-health. We therefore investigated the role of the home environment in Ugandan children taking into account the frequent infections and extreme poverty in which they lived.

**Methods: **Cognitive abilities of 163 5-year-old children were assessed. Home environments of these children, their health status and family socioeconomic status (SES) were assessed respectively using the EC-HOME, anthropometry and illnesses, and traditional SES measures. Structural equation analyses compared five models on the influence of the home environment, SES, and child health on the cognitive scores.

**Results:** The model in which the home environment mediates the combined influence of SES and child health on cognitive performance showed a particularly good fit to the data compared with the four alternative models, i.e. those in which the HOME, SES and health independently influence cognitive performance.

**Conclusions: **Home environments providing cognitive stimulation can enable children to overcome effects of major adverse life experiences on cognitive development.

## Introduction

The home environment is considered to be of paramount importance in neurocognitive development, especially in the first years of life when children’s experiences are predominantly dependent on what is provided by their parents. The home environment comprises physical (e.g. household possessions, play materials) and social (e.g. parent-child interactions, family size, and structure) components, which, if favourable, provide psychological stimulation and support necessary for optimal development of early cognitive skills and these in turn predict their education and employment success later in life. Evidence for the role of the home environment comes from observational as well as interventional studies ( Andrade *et al*., 2005; Bradley *et al*., 1989; Dobrova-Krol *et al.*, 2010; Grantham-McGregor *et al.*, 1994; Klein, 1991; Klein & Rye, 2004; Laude, 1999; Marques dos Santos *et al*., 2008; Ramey & Ramey, 1998; Richter & Grieve, 1991; Roberts & Barnes, 1992; Waber *et al*., 1981).

Parental responsivity, frequent contact, consistent provision of care and a variety of play materials correlate strongly with cognitive development ( Andrade *et al*., 2005; Bradley *et al*., 1989; Laude, 1999). Family care, even when it is of compromised quality, is more favorable for children's development than institutional care ( Dobrova-Krol *et al*., 2010). In general, the social (parental warmth) and contextual exposures within the home environment provide opportunities for children to learn language and other cognitive skills useful for everyday learning, until they start schooling and beyond ( Roberts & Barnes, 1992). The home environment therefore plays a critical role in laying the foundation for basic cognitive capacities on which school and other external environments will build.

Evidence indicates that the quality and impact of the home environment vary with family poverty, parental education, and other socio-economic factors ( Andrade *et al*., 2005; Bradley *et al*., 1989; Coscia *et al*., 2001). Parents living in poverty are unable to provide stimulating materials (e.g. toys, and books) for their children and are often stressed and use harsh punishments to discipline their children. Repeatedly, the effects of socioeconomic status (SES) on cognitive function have been found to be mediated by the home environment ( Coscia *et al*., 2001; Marques dos Santos *et al*., 2008), which is consistent with Bronfenbrenner’s (1979) ecological systems theory, in which SES influences development through the more proximal family environment.

Further, the influence of the home environment on cognition has often been reported to be compromised by ill-health, ( Coscia *et al*., 2001; Dobrova-Krol *et al*., 2010; Marques dos Santos *et al*., 2008; Sarsour *et al*., 2011). For instance, the effect of the home environment was weaker in HIV-infected children than in controls, possibly because of reduced activity in the sick children, but also discrimination of these children by family members, blunting the effect of the home environment ( Dobrova-Krol *et al*., 2010). Occasionally, severe ill-health may enhance the health-cognition relationship. Coscia *et al.* (2001) reported this in children who were in the advanced stages of HIV disease when compared with those in earlier stages of the disease. In summary, child stimulation in the home environment is constrained by its complex relationship with socio-economic and health factors. Thus measuring the home effect should take these factors into account.

**Figure 1.**
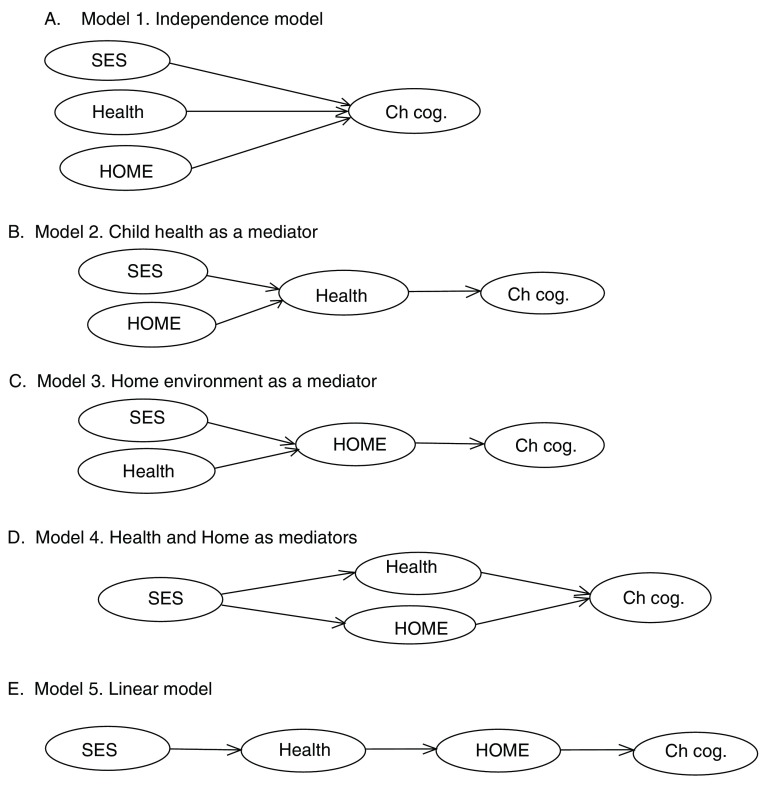
**A**. Model 1, in which child health, SES and home environment are hypothesized independently to influence cognitive function; **B**. Model 2 in which child health is hypothesized to mediate effects of SES and the home environment on cognitive function; **C**. Model 3 in which the home environment is presumed to mediate effects of SES and child health on cognitive function; **D**. Model 4 where the child’s health and home environment each mediate the effects of low SES; **E**. Model 5 in which a series of mediations are seen, starting with SES, through child health and home environment to cognitive function.

The current study aimed to measure the impact of the home environment on cognitive function in a sample of Ugandan children, taking into account possible direct and indirect influences of frequent infections and the extreme poverty which these children experience. On the basis of the available literature, we hypothesized alternative models describing the relationship between the home environment, SES, child health and cognitive function. In this paper, we consider five models, which are described below and shown in Figure 1.

The home, SES and child health status are often taken as having competing influences on cognitive function and, as a result, there is a large body of evidence for the distinct effects of each of these on cognitive outcomes ( Bradley *et al*., 1989; Ramey & Ramey, 1998; Walker *et al.*, 1991). This relationship is represented as Model 1 in Figure 1. However, the home environment, SES and health factors may interact in different ways to influence a child’s development. This may be through mediation of the effects of one factor by another or by modification of its relationship with cognitive function. Mediation/modification effects have been demonstrated in studies among HIV-infected children ( Coscia *et al*., 2001; Dobrova-Krol *et al*., 2010). Both of these studies show that HIV may negatively modify the effect of the environment, but, in advanced stages of the disease, the effect may be positive ( Coscia *et al*., 2001). SES as a broad construct (parental education, occupation and family income) has been found to be associated with cognitive performance ( Berkman *et al.*, 2002; Humphreys & Grencis, 2002; Jukes *et al*., 2002; Marques dos Santos *et al*., 2008; Ravara & Cunha, 2016). However, studies indicate that the relationship between SES and cognitive function might be mediated by factors within the home environment, including play materials, parenting style and the physical environment ( Coscia *et al*., 2001; Sarsour *et al*., 2011; Tong *et al.*, 2007). From these studies, two alternative models are hypothesized: one in which health status mediates effects of SES and the home environment ( Figure 1, Model 2), and another where the home environment mediates effects of health and SES ( Figure 1, Model 3). 

Studies have consistently demonstrated an association between SES and child physical health. Children living in poverty are at a higher risk of low birth weight ( Rouse *et al.*, 2015), child mortality ( Shigeno *et al*., 2014) and poor nutritional status ( Braissant *et al*., 2015; Liu *et al.*, 2015). These children consequently suffer poorer neurodevelopmental outcomes than those who are from wealthier families ( Kordas *et al*., 2009). Given that SES also contributes to differences in the quality of the home environment ( Bradley *et al*., 1989), it is possible that the effect of SES on a child’s intellectual ability is mediated by the child’s health and the home environment. On the basis of this notion, a fourth model (Model 4, Figure 1) can be hypothesized, in which SES is moderated both by the home environment and the health of the child and each separately affects cognitive function. 

In contrast to the aforementioned models, some findings have implied a linear sequence of influences leading to prediction of the child’s cognitive function. A prior study showed that SES was related to health status, which in turn fed into the home environment to ultimately affect cognitive function ( Marques dos Santos *et al*., 2008). Thus, building on Bronfenbrenner’s ecological systems theory ( Bronfenbrenner, 1979), an alternative model (Model 5) predicting a clearly linear series of influences from SES (most distal and lasting), through the child’s health from birth, then the current home environment (most proximal) to cognitive functioning in childhood is proposed. Figure 1 presents the five contrasting models proposed above.

Apart from the few studies in low-income settings ( Marques dos Santos *et al*., 2008) evidence for the role of the home environment is largely from studies of families in the West, where the effect of the home environment is less likely to be significantly influenced by major adversities. Western samples have included participants with low SES, but these did not have the level of poverty, illiteracy, malnutrition, infection, and mortality rate that predominate in sub-Saharan Africa, the Indian subcontinent and developing countries within Asia.

These adversities make the sub-Saharan region an excellent context in which the role of the home environment on development may be compromised by these factors but such contexts have not been studied.

Using a sample of children from a low-income setting who suffered frequent infections in early childhood, we aimed to (i) to assess scores on the Home Observation for Measurement of the Environment (HOME) scale of children living in typical poverty with typical infections; and (ii) to measure the relative impacts of the child’s social circumstances, health status and home environment on cognitive ability by comparing the models shown in Figure 1 to identify which best describes the relationship between the three key exposures (home, SES and health) and cognitive performance.

## Methods

### Design and participants

This research was conducted within the larger birth cohort that investigated effects of worm infections and their treatment on responses to immunizations, incidence of childhood infections and of allergic diseases in children, and on cognitive outcomes ( Elliott *et al*., 2007). Between January 2010 and December 2012, families participating in the parent study and residing in Entebbe municipality, Uganda, and the surrounding villages were invited to take part in this study. A sub-group of 163 participants (80 males, 49%) of mean age, 5 years, 2 weeks, was selected from among the 870 5-year-old participants for whom cognitive testing had been completed to assess outcomes of anthelminthic treatment in children ( Ndibazza *et al*., 2012). The home observations were introduced after cognitive testing had begun. Children who participated in cognitive testing (at age 5 years) during this time, and were eligible, were enrolled as they came in. Children were eligible for home observations if they were 5 years old plus or minus 2 weeks, were participating in the EMABS study, had completed the cognitive assessment and were residing within Entebbe Municipality and Katabi sub-county, and their parents were willing to participate in this part of the study. When recruitment started, every parent who brought the child for the 5 year visit was approached about this study and if they were willing and the child was eligible, they were recruited. Recruitment was stopped when the desired number of participants was obtained. A previous study with a sample size of 89 generated enough power to give a significant result ( Bangirana *et al*., 2009); thus, a sample size of 200 children was estimated to be sufficient for this question. However, a sample size of 163 was achieved. A total of 17 children missed the HOME assessments, hence the data reported here are on 146 children.

### Measurements

***The home environment*.** The home environments of participants were measured using the HOME scale ( Caldwell & Bradley, 1984), which was adapted and translated to suit the study setting ( Supplementary File 1). The HOME inventory was developed to measure the quality of stimulation and support available to a child in the home environment; it assesses various aspects of the home environment, including emotional and verbal responsiveness of the mother, use of restriction and punishment, organization of physical and temporal environment, provision of appropriate play materials and games, mother’s involvement with the child, and opportunity for variety in daily stimulation. The early childhood HOME scale, which was adapted for use in this study, covers ages 3–6 years and assesses various aspects of the home environment using eight subscales (the physical environment, learning materials, language stimulation, responsivity, academic stimulation, modelling and acceptance). The HOME has been used world-wide and has been demonstrated to exhibit stable validity across a diversity of cultural and socio-economic contexts studied ( Bradley *et al*., 1989; Mitchell & Gray, 1981; Mundfrom *et al.*, 1993; Plomin *et al.*, 1985; Stevens & Bakeman, 1985). However, some items have been reported to be inappropriate for certain cultures outside the US ( Caldwell & Bradley, 1984), so it was initially piloted on 20 families and minor modifications were made to suit it to the study population. A full description of the how the HOME was adapted, including the pilot, is provided in Supplementary File 1.

***Motor and cognitive testing*.** Participants were assessed on motor and cognitive functions using measures that were previously adapted and translated for Ugandan children ( Nampijja *et al*., 2010), and four additional measures of executive function that were added later, which are described in Supplementary File 2. The assessment battery comprised measures of motor ability, general intellectual ability and executive function (i.e. working memory, inhibition, mental flexibility, attention and planning). These domains have been implicated in previous studies to be sensitive to effects of worm infection, and respective measures have shown adaptability across various contexts. The measures included in the battery were: coin box and balancing on one leg (motor ability); block design and picture vocabulary scale (general cognitive ability); sentence repetition, verbal fluency, counting span and running memory for working memory; tap once tap twice (henceforth referred to as the ‘Tapping task’: inhibition); Wisconsin card sorting task (mental flexibility); Picture Search (selective attention); and Tower of London (planning), (see Ndibazza *et al*., 2012).

***Health and social exposures*.** Apart from the home environment (key exposure) and cognitive performance (main outcome), variables specifying child health and social processes were examined to complete the conceptual framework represented in Figure 1. In terms of child health, we recorded antenatal and delivery information, childhood illness episodes (malaria, diarrhoea, upper and lower respiratory tract infections, measles, HIV status, worm infections), child’s nutritional status (height, weight, haemoglobin levels). For information on socioeconomic circumstances, we included the child’s birth order, number of siblings, mother’s age and marital status, family size and composition, and measures of family wealth, including mother’s and father’s education, occupation, income, and household SES. Household SES was rated on a six-point scale based on possession of items such as a bicycle, television, phone, bed, etc. In addition to the home environment, we measured the ‘out of home’ environment, particularly school attendance, since by age 5 years Ugandan children have been enrolled in preschool. We collected information on whether the child was attending any form of school (day-care, preschool or primary), for how long they had attended and how many hours were spent there.

### Ethical approval

The study was approved by the Science and Ethics Committee of the Uganda Virus Research Institute (Ref. GC 127/11/08/20) and the Uganda National Council for Science and Technology (Ref. SS 2262). Informed written (or witnessed thumb-print) consent was obtained from parents or guardians of all eligible children.

### Data reduction

Data were analyzed in SPSS version 12. With several measures assessing each of the complex constructs that we wished to explore, the first task was to examine whether and how the four global constructs (HOME, health, Social and Economic Status (SES), and cognitive ability) could be reduced for further analysis. We used Cronbach’s alpha as an initial guide in preparation for the modelling, in which the validity of factor loadings could be confirmed.

 The eight HOME measures were examined first and found to correlate with each other. The measures seemed to show reasonable consistency (the exception was Acceptance which was dropped). To confirm this we ran a Confirmatory Factor Analysis using the package AMOS. This showed an acceptable fit (root mean square error of approximation (RMSEA) = 0.04) and each of the manifest variables contributed significantly to the latent factor (p < 0.001). It was thus decided to treat these variables as a uniform construct in preparation for the main analyses.

Secondly, we examined the relationships between seven variables used to assess SES: maternal and paternal educational attainments, maternal and paternal occupation, marital status, household possessions and family income. On the basis of correlations, the best combination of measures involved four of these (paternal occupation, maternal education, family income and household possessions) and these factors are commonly taken to be measures of SES.

Thirdly, we constructed a measure of the child’s health. This took into account the child’s weight and height, and we calculated mean of the standardised scores from these two measures. We also derived a standard measure drawn from the number of key illnesses experienced (averaged scores of number of diarrhoea episodes, lower tract infections and malaria bouts). The rationale for this was that at this age and in this culture weight and height are markers of good health, while illnesses are a marker of ill health, as discussed in the background. For the preliminary analyses we constructed a measure of ‘physique ‘(height + weight/2) and subtracted a measure of the sum of the three key illnesses, using standard scores, to provide a measure of child health.

Fourthly, we constructed a measure of cognitive functioning from the mean of the standardized scores from the nine test measures. We found (following exploratory factor analysis and an attempt to construct separate measures of verbal and nonverbal performance) that a single scale of seven of these items produced the most coherent overall measure. Correlations between each HOME dimension and cognitive measure were examined.

### Comparison of identified models

In order to test the coherence of the measures comprising the four constructs (SES, Child Health, HOME and Child Cognitive Performance) and compare the relative fit of the five models depicted in Figure 1, we constructed structural equation models (SEMs) using the AMOS statistics package ( Brooker *et al.*, 2006). Note that the correlation between SES and Child Cognitive Performance in Table 5 was not significant so we examined whether the other variables (Child Health and HOME) mediated the link between these two factors. The models also allow us to test the possible ways in which child health and the home environment might channel SES influences or mutually influence child cognitive development. We identified ‘illness’ as a negative value and linked all the individual measures with their designated latent variables. There were a few missing values for each variable (see Table 4) and these were accounted for using the full information maximum likelihood estimation procedures in AMOS based upon a missing at random assumption. We used the following indices of the fit of the models: [1] likelihood ratio chi-square: while not recommended for SEMs with small samples ( Xie & Fu, 2012), it is included in this analysis for completeness; [2] Parsimony Comparative Fit Index (PCFI) is recommended to be a useful index of fit ( Boivin *et al*., 1993), even though there are not agreed cut off levels for an acceptable model; [3] Root Mean Square of Approximation (RMSEA), as this provides 90% confidence intervals and agreed upon levels of model fit with RMSEA < 0.1 as ‘acceptable’ and RMSEA < 0.06 as ‘good’ ( Boivin *et al*., 1993); [4] Akaike Information Criterion (AIC) to allow model comparison with lower values showing relative better fit.

## Results

### Participant characteristics

Mean birth weight, weight, height and haemoglobin level of the subgroup were found to be similar to those of the rest of the main cohort. In keeping with the larger sample, 6.8% were underweight (weight <13.8 kg), 12.5% were stunted (height <95 cm), and 5% were anaemic (Hb <10 g/dl). During the period from birth to 5 years of age, 27% of these children had suffered more than two episodes of malaria, 65% had had two or more bouts of diarrhoea and 98% had suffered frequent upper respiratory tract infection, often occurring as co-infections (so we did not examine this variable further). Lower respiratory infections were less common. Thirteen children were exposed to HIV infection *in utero*, three of whom were HIV-positive. These data are summarized in Table 1.

**Table 1.** Child health characteristics.

Child’s nutritional parameters						Maternal factors		Childhood illnesses					
	Bwt, kg	Height, cm	Weight, kg	Muac, cm	Hb, g/dl	Mother’s age, years	Family size, people	Events	Malaria, n (%)	Diarrhoea, n (%)	LRTI, n (%)	URTI, n (%)	HIV *, n (%)
Mean	3.31	101.64	16.32	16.74	11.99	24.86	5.70	none	76 (52.8)	24 (16.7)	106 (73.6)	nil	122 (90.4)
SD	0.47	4.96	1.88	1.13	1.32	6.37	1.93	1	29 (20.1)	26 (18.1)	26 (18.1)	2 (1.4)	13 (9.6)
Min	1.00	89.5	12	14.00	6.80	15	2	2+	39 (27.1)	94 (65.3)	12 (8.3)	142 (98.6)	
Max	4.2	117	21.4	19.20	16.80	47	19						

*Perinatal exposure to maternal HIV infection. Bwt, birth weight; Muac, mid upper arm circumference; Hb, haemoglobin level; LRTI, lower respiratory tract infection; URTI, upper respiratory tract infection.

Turning to the social circumstances of participants, parents of these children were of variable education status, although the majority (123; 91.9%) did not go beyond secondary education. Mothers were less educated than fathers. Parents mostly engaged in a range of unskilled jobs, 84.8% earning less than 30,000 Uganda shillings ($12) per month. In general, the sample exhibited social circumstances of an extremely poor population, and one in which childhood illnesses were common ( Table 2). All raw data are available on the LSHTM Data Compass ( Nampijja, 2018)

**Table 2.** Family socio-economic characteristics. Characteristics of participating children and their parents were similar to those of the parent sample from which they were selected.

Parental education			Parental occupation			Household possessions		Mother’s income (*U shs)		Marital status		Parity	
Level	Mother N (%)	Father N (%)	level	Mother N (%)	Father N (%)		N (%)	Amount	N (%)	status	N (%)	number	N (%)
None	3 (2.2)	4 (2.3)	None	8 (5.9)	2 (1.6)	1	12 (9)	<30K	112(84.8)	single	22 (16.3)	1	31 (23)
Primary	70 (51.9)	33 (21.8)	Farmer	5 (3.7)	14 (11.2)	2	6 (4.5)	30–60K	10 (7.6)	married	106 (78.5)	2–4	75 (55)
Senior	50 (37.0)	73 (45.3)	Unskilled	9(6.7)	50(40.0)	3	37 (27.8)	60–100K	2 (1.5)	widowed	1 (.7)	5+	29 (21)
Tertiary	12 (8.9)	14 (12.4)	Bar	81 (60.0)	-	4	39 (29.3)	>100K	8 (6.1)	divorced	6 (4.4)		
NA		29(18.2)	Business	19 (14.1)	16 (12.8)	5	29(21.8)						
			Student	3 (2.2)	3 (2.4)	6	10 (7.5)						
			Professional	10 (7.4)	40 (32.0)								

SES = socio-economic status; K, 1000 Uganda shillings; 2500 Ugandan shillings = $1 (the exchange rate at time time).

### Profiles of the HOME subscales

Descriptive statistics (mean score, standard deviations, minimum and maximum scores) of scores on the eight subscales of the HOME were examined first to see if the tool works in a Ugandan setting. Scores on all the subscales except Acceptance had normal distributions, hence the HOME inventory was appropriate for this sample. There was also sufficient variability within the sample making the data suitable for comparisons within the sample. These descriptive data are shown in Table 3.

**Table 3.** Descriptive statistics for scores on the HOME.

HOME subscale	N	Min	Max possible	Mean	Skewness	Kurtosis
Learning Materials	157	1	13(13)	6.75(2.14)	.45	.10
Language	145	2	7(7)	4.99(1.35)	-.35	-.44
Physical Environment	161	0	7(7)	3.58(2.12)	-.02	-1.17
Responsivity	156	0	8(8)	2.22(1.93)	1.27	1.17
Academic Stimulation	161	0	4(4)	2.88(1.04)	-1.17	1.14
Modelling	111	0	5(6)	2.06(1.20)	.17	-.57
Variety	155	1	7(9)	3.59(1.48)	.28	-.58
Acceptance	145	1	7(7)	5.77(1.19)	-1.27	1.90

**Table 4.** Descriptive statistics for motor and cognitive scores.

Domain	Measure	N	Min	Max (max possible	Mean	s.d
Motor Function	Coin Box	144	5.5	16.50 (20)	9.94	1.54
	Balancing on one Leg	142	2	52 (60)	14.88	11.16
General cognitive ability	Block Design	142	1	51(16)	7.75	3.06
	Picture Vocabulary Scale	142	8	23 (24)	17.07	3.25
Working memory	Sentence Repetition	141	8	31(34)	19.88	4.03
	Verbal Fluency	143	0	32 (NA)	14.45	7.79
	Running Memory	142	3	20 (20)	12.15	2.85
	Counting Span	141	0	7 (8)	3.60	2.11
Selective attention	Picture Search	145	.67	7.33 (10)	4.01	1.32
Mental flexibility	Wisconsin Card Sort	145	0	12 (12)	5.80	3.87
Inhibitory control	Tapping Task	145	0	12 (12)	5.17	4.69
	Shapes Task	143	0	12 (12)	5.81	3.64
Planning	Tower of London	136	0	10 (10)	2.46	3.07

NA, not applicable.

**Table 5.** Correlations between the four composite measures.

	HOME	Cognitive performance	Child health
Cognitive Performance	0.42 **		
Child health	0.24 *	0.32 **	
SES	0.47 **	0.14	0.32 **

**p<0.01 (2-tailed); *p< 0.05(2-tailed).

### Descriptive statistics of scores on the motor and cognitive measures

The sample exhibited variability in ability on the measures of motor and cognitive function and they showed normal distributions: hence the performance data were suitable for parametric tests. Two measures (balancing on one leg and Tower of London) were moderately skewed, but all skewness and kurtosis values were within the range -2 to +2, so we did not transform any variables. Distribution of performance on the various measures is summarized in Table 4.

Correlations between each HOME dimension and cognitive measure were examined. Table 5 presents the correlations between the four measures constructed to assess the factors depicted in Figure 1. It shows that all these measures were significantly related to each other, with the exception of the link between SES and the child’s cognitive performance.

Table 6 reports these figures and the right hand column summarizes the non-significant regression weights, where appropriate, as these show how models can be made more parsimonious (and by implication whether individual pathways fit). Table 6 also includes the crucial parameter estimates in the best fitting model.

**Table 6.** Comparisons between the structural equation models.

Model	χ ^2^ (df)	PCFI	RMSEA (90% CI)	AIC	Non-significant parameter estimates in Models 1-3 compared to those in Model 5				
					Structural pathway	Estimate	s.e.	c.r.	p
1a Independence	286.24 (169)	.57	.069 (.055-.083)	408.24	SES- Child cogn.	.09	.25	-1.3	.72
1b Independence (with covariates)	246.41 (166)	.63	.058 (.042-.072	374.41	HOME-Child cogn.	.64	.42	1.53	.13
					SES- Child cogn.	-.7	.7	-.99	.32
					Child health- Child cogn.	3.74	2.7	1.3	.17
2. Child health as mediator	248.40 (167)	.64	.058 (.042-.073)	374.40	SES-Child health	.03	.08	.43	.66
3. Home environment as mediator	246.41 (166)	.63	.058 (.042-.072)	374.41	Child health-HOME	.88	1.06	.83	.41
					HOME-Child cogn.	.64	.42	1.53	.13
4. Home and health mediating SES effects	255.23 (168)	.68	.06 (.044-.074)	379.23	HOME-Child cogn.	1.40	.59	2.40	.01
					Child health – Child cogn.	-4.45	3.54	-1.26	.21
5. Linear model	253.77 (169)	.64	.059 (.043-.073)	375.77	SES-Child health	.23	.06	3.68	<.001
					Child health-HOME	3.89	1.87	3.28	.001
					HOME-Child cogn.	.87	.24	3.62	<.001

χ ^2^, likelihood ratio chi-square; PCFI, comparative fit index (>0.9 suggests adequate fit but see qualification in text); RMSEA, root mean square error of approximation (<0.06 suggests ‘good’ fit); AIC, Akaike’s information criterion (lower values suggest better models); c.r., critical ratio

As Table 6 shows, all the models fitted, as assessed by the key index of model fit, RMSEA < 0.1. SEM 1a is the ‘Independence’ model depicted in Figure 1a, in which the three explanatory variables are hypothesized to be unrelated to each other. This model did not fit as well as the others. In addition, here as in most of the following analyses, one of the three key structural paths, from SES to the dependent measure, Child Cognitive Performance, was non-significant (see Table 6, right-hand column). The standard procedure of removing non-significant regression weights would destroy a crucial feature of this model, so we must reject it. Adding the covariances between the three explanatory variables (essentially a regression model: Table 6, 1b) made the model fit better (RMSEA < 0.06), although none of the three key structural paths was significant. Models 2-5 showed the same acceptable levels of fit (indeed they were almost indistinguishable) but in the various mediation analyses (Models 2-4) there were again key paths that were non-significant (see Table 6, right-hand column). Including/excluding direct links between the two left-hand measures and child cognitive performance (i.e. examining the full mediation links) made no difference to the significance of the model or the structural pathways. Only the linear model (Model 5) showed both acceptable fit and significant links between the variables.

**Figure 2.**
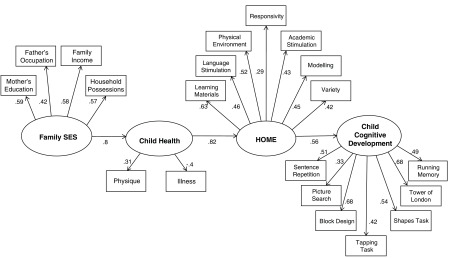
The model shows the regression weights for the links with manifest and latent variables comprising SES, child health, home environment and cognitive function.

Figure 2 summarizes Model 5, although for the sake of simplicity it excludes the error and disturbance values (all showed acceptable links with the associated manifest or latent variable). As with all the other models, the RMSEA showed a good overall fit and all the measures significantly fitted their associated latent variables. However, unlike models 1-4, the crucial pathway between SES and child health (p < 0.001), child health and the HOME factor (p < 0.001) and the HOME and the child’s cognitive performance (p = 0.001) were strongly associated. 

We examined the possibility of dividing the HOME measures into two subtypes (provision for the child vs. interaction with the child), but a confirmatory factor analysis did not produce a sufficiently strong enough factor structure (RMSEA = 0.16) and we did not proceed with this analysis. However, Model 5 seems to fit the data very well. Assuming that SES was consistent from the child’s birth, the child’s health measure recorded illnesses from birth to the age of 5 years and the cognitive development measures were recorded at that age, there is a time sequence built into the model in Figure 2.

## Discussion

This study investigated the relative influences of the long-identified connections between the home environment, child health and socio-economic status on a 5-year-old’s cognitive function in children in very low-income settings ( Sternberg *et al.*, 1997). In line with our predictions, the data showed significant correlations among these three variables, and (with the exception of SES) these correlated with a wide range of cognitive tests (see Table 5). The relationship between the HOME score and cognitive performance was typical, given that many studies show correlations ranging between r= 0.2 and r= 0.6 ( Michaelsen, 1985; Sayed *et al*., 2005). Bradley’s ( Michaelsen, 1985) recent theoretical analyses of the means by which caregivers channel social and biological processes of the child’s development suggests that structural equation modelling and similar multivariate approaches will enable us to examine the complexities of the many variables that may influence child outcomes. Using SEMs, we tested a range of competing hypotheses about the nature of the interactions between these factors. While most of the models showed a good fit to the data, as assessed by RMSEA, only the one depicting a linear pathway from SES, through child health to HOME, showed significant links at all levels ( Table 6 and Figure 2).

The best fitting model ( Figure 1, Model 5) bears some similarity to that of Marques dos Santos *et al*. (2008), who showed close connections between SES, health and the home environment. Their layered multiple regressions tested a different direction of causality from SES, through material and psychosocial stimulation to a variety of ‘child characteristics and health’. Our findings do not replicate all the pathways that they found to be related. However, the two studies may not be incompatible, as both suggest that the means by which parents structure the child’s learning and psychosocial resources may provide a channel through which life experiences and the parent’s and child’s individual propensities can affect the latter’s cognitive function. The close proximity of the home environment with cognitive development in our model gives it a critical role of protecting against the negative impact of ill-health and adverse SES. This is consistent with recent analysis of 117,000 households by Bornstein *et al*. (2015), which found that the resources available at home make up three-quarters of the link between SES factors and the child’s development, and with findings of earlier studies in Ugandan and Kenyan children that showed no direct relationship between SES and psychomotor development ( McCall *et al*., 2014; Sayed *et al*., 2005). The focus in this study, children living in poverty and susceptible to frequent and serious illness, accentuates the importance of the home environment when such threats are regular and intense.

The data in Model 5 suggest that within this setting exploring the link between family SES and the child‘s development, the HOME seems to be an important factor that links SES and child outcomes. It is not just the child who is susceptible to malaria, but other family members too, who contribute to the social and physical aspects of the home environment. Family health is bound to have an effect on the home environment and this might explain both the failure of the two-factor model in confirmatory factor analysis and the pivotal role of the HOME in model 5.

The data analyzed in this study were cross-sectional. Longitudinal research has long found continuities in the ways in which the home environment is managed by parents, but this research is less clear in terms of the lasting effects of the HOME measure, for example from infancy through to the school years ( Bradley *et al*., 1988; Bradley *et al*., 1989). Future analyses should examine whether the pattern depicted in Figure 2 is shown in SEM analyses in which data are collected longitudinally. Such analysis with a larger sample might also show more specific relationships between aspects of the home environment, such as social stimulation and particular developmental outcomes like social cognitive skills ( Alderman *et al.*, 2006). The linear sequence of the variables in Model 5 may be a reflection of timing for these factors (SES, health and HOME) in the first 5 years of life; SES tends to be longstanding and the child’s health measures record events and experiences over the period of 5 years, while the HOME is the most current and is the most proximal influence that we measured. This suggests a sequence of influences, as can be seen in Figure 2. However, it is important to know how such effects may change over time, and discerning the series of influences that is represented is only possible using longitudinal studies. In recent years researchers have also shown the importance of extending the focus to include the child’s own contribution, particularly genetic influences, and extending the boundaries of the role of social processes ( Michaelsen, 1985). Particularly outside the Western context, the child’s physical and social resources extend beyond the household to include neighbours and members of the local community ( Stoltzfus *et al*., 2001) and further work in contexts like Uganda should be attentive to such influences.

## Conclusion

Overall, the data indicate that even where resources are limited and children are exposed to regular infections and diseases like malaria, their development can still be promoted by ensuring that they are provided with a stimulating home environment (especially modelling and academic stimulation). Hence a slight modification of the slogan seems warranted: “a healthy mind needs not only a healthy body but also healthy (stimulating) environment”.

## Data availability

Access to the EMABS Home Observation data is restricted, in order to protect participant confidentiality and comply with the study’s ethical commitment to ensure data is used for legitimate research. It will be provided for use in ethically-approved research, subject to a commitment that it will be held securely and used for the approved purpose only. Applicants should complete a data request form at https://doi.org/10.17037/DATA.00000792 ( Nampijja, 2018) and submit it for consideration by the study data access committee. If the proposed use is compatible with the study’s ethics permissions, the applicant will be asked to sign a Data Sharing Agreement and subsequently provided with the dataset.

## Supplementary material

Supplementary File 1. Adaptation and pilot of the HOME.

Supplementary File 2. Adaptation and translation and piloting of extra measures of executive function.
