# Supplementary material for: The role of the home environment in neurocognitive development of children living in extreme poverty and with frequent illnesses: a cross-sectional study
Source: Wellcome Open Res. 2018 Dec 3;3:152. [Version 1] doi: 10.12688/wellcomeopenres.14702.1 (PMC6338129; doi:10.12688/wellcomeopenres.14702.1)
Supplement: Supplementary file 1 [file wellcomeopenres-3-16011-s0000.tgz › 650e217d-8d81-4768-ae73-622d48e687ef_Supplementary_File_1.docx]

**Supplementary file 1: Adaptation and pilot of the HOME**

The first step in adapting the HOME was to determine the suitability of each of the items contained in the tool for our setting since the version that was used (i.e. the Early Childhood HOME) was standardised based on data collected from the US where culture, and child rearing practices and socioeconomic status are different from those of the study population. Next, items in the HOME were modified and this was based on results of the first step. Modifications included adding new items, changing existing ones or even dropping those that were inappropriate to the target population. The final step involved assessing the suitability of the adapted version of the HOME by piloting it on a sample of homes. Details of the adaptation procedure follow shortly. As indicated by Vogt, King, & King (2004), while developing a measure, the local community should be consulted about the appropriateness of the items that comprise it. In particular, Vogt and colleagues emphasize the use of focus group discussions for obtaining constructive feedback from the community. Based on this recommendation, focus group discussions were used to identify the relevance of each of the items in the HOME and which items had to be added. Mothers participating in the EMABS study and community field workers volunteering in the same study were approached to participate in these discussions. Only mothers whose children had already completed their 5^th^ annual visit were selected to participate in the focus group discussions. This criterion was important because we did not want mothers who would later be included in the main home observation to have prior knowledge of the items we were interested in from the home observation. Four focus groups of participating mothers were generated each comprising 10 members. Another block of four focus groups comprising field workers (10 in each) were used. Of the 10 fieldworkers included in each focus group, two were men. In this setting, mothers are traditionally more involved in the day to day caring of children than fathers even in the case of single parenthood. However, occasionally, marriages have broken and the children have been left under the care of their father. It was therefore important that the views of the fathers were also represented although it appeared that their ideas did not differ from those of the mothers.

Using the subscales and the items listed in the EC- HOME, questions were formulated and used the in the focus group discussions to generate play items/child-parent-behaviour that are common in this setting. Discussions were chaired and moderated by the author of this manuscript, who briefed the members about the objective of the exercise and encouraged them to give responses based on real life experiences in their homes, at the neighbours, friend or local community in general. Discussions commenced with a general question “What do mothers (fathers) and their children do at home?” This was followed by more specific but still open ended questions like “How do mothers interact with their children?” Responses were recorded by a facilitator as he also cross-checked with the list of items in the original version of the HOME. There was generally an overlap between activities generated from the discussions and the items listed in the original version of the HOME. However, a few of the items were found to be uncommon; these included reading or buying magazines, child possession of own radio or music instrument, mothers kissing their children, and reading daily newspaper. These items were not deleted but their scoring was adjusted. New items such as play materials not owned by the child, whether the child was attending a play group and/or Sunday school were added. The meetings lasted between 2-3 hours depending on how active the members in the group were. Mother- focus group meetings were alternated with field worker focus groups meetings. After the first four focus group discussions, the information generated was reviewed and used to make initial changes to the HOME. At this level all the original items in the HOME were retained and new ones were added. The new version of the HOME was then piloted on 15 homes. The initial piloting phase revealed that the items were appropriate but many of items had zero variability which was mainly because of the scoring. Based on results of the initial pilot, more changes were made to the instrument, in particular adjustments to the scoring of items were made to allow more variability. New items were also added. A second round of focus group discussions was then conducted to evaluate items in the new version of the HOME. Changes to the HOME were dependent on the item in question. In some of the questions the scoring was adjusted, for example for number of child appropriate books owned, having at least one book was given a score. In some questions local examples were inserted: For example in the question asking for puzzles, we included examples of locally made puzzles e.g. luddo. Further, extra questions were added to the 58 that constitute the standard HOME. Such questions like whether the child played with play toys that allow pretend play even if they did not own them; whether the child played with puzzles even if they did not own them; whether a child attended a play group; if they attended Sunday school. Altogether 10 questions were added. The resultant version was piloted on 12 homes. Data from the second pilot showed marked improvements in the variability within each item. The final version comprising 68 items which were either observed or the mother interviewed about them was compiled and used to assess the study participants. Similar to the original HOME, items were scored plus if present or minus if absent and the total number of pluses in each subscale was counted.
