# Supplementary material for: The role of the home environment in neurocognitive development of children living in extreme poverty and with frequent illnesses: a cross-sectional study
Source: Wellcome Open Res. 2018 Dec 3;3:152. [Version 1] doi: 10.12688/wellcomeopenres.14702.1 (PMC6338129; doi:10.12688/wellcomeopenres.14702.1)
Supplement: Supplementary file 2 [file wellcomeopenres-3-16011-s0001.tgz › cdc130fa-f5b2-4450-9474-3cd81f7ef2e0_Supplementary_File_2.docx]

APPENDIX 2: Adaptation and translation and piloting of extra measures of executive function

Four extra measures of executive functioning added to the original battery were: two measures of working memory (Counting Span and Running Memory), one measure of inhibition (Shapes Task), and one measure of planning (the Tower of London-TOL). Each measure was modified to suit these children. The new versions were piloted on 25 five-year-old children recruited from the study area. A detailed description of the four extra measures follows.

*Counting Span.* The task was originally developed by Case, Kurland, & Goldenberg (1982) to measure total processing and storage space (*M-space*) for working memory in children aged 6-12 years. Similar versions (paper card as well as computerized) have successfully been used for assessing children on working memory span (e.g. Towse & Hitch, 1995; Towse, Hitch & Hutton, 1998). A simpler modification of the Counting Span task was constructed and used to assess working memory in our participants. It consists of eight arrays of cards which the child counts and has to note the picture on the target card (first card). At the end of counting the cards in each array, the child is required to recall and say the picture on the target card. The first trial comprised an array of three cards, the second trial four cards and so on up to the eighth trial which had 10 cards. The pictures used in this study were all hand drawn black and white line drawings. They were of relatively equal in size. The experimenter initially reviews all the pictures on the cards with the child to ensure that the child knows all their names. Trials are preceded by a demonstration example. One point is awarded for a correct response and a zero for an incorrect response and feedback is given for each trial done. There is therefore a maximum possible score of eight on this measure. Using this paradigm, novel pictures were compiled; these were selected based on the objects that are found in the environment and which young children are familiar with. Children were asked to name the items in Luganda; however responses given in English were acceptable provided they were correct. The shapes and pictures were piloted and those that were found to be strange (difficult to name) were replaced with more familiar items.

*Running Memory.* This measure was adapted from a running memory task version (Kramer, Larish & Strayer, 1995) to measure working memory span. The version devised for this study consisted of 10 strings of common of unrelated two- syllable Luganda words which were read out to the child at a regular speed of one word per two seconds. The child is required to repeat each string verbatim immediately without changing the word order, omitting or inserting a word. The Running Memory task has often been used as a measure of working memory although some psychologists argue that this and similar tasks such as Digit Span Forward, Word Repetition, and Sentence Repetition simply measure storage capacity (e.g. Hutton & Towse, 2001). Two points are awarded if no error is committed; one point if not more than two errors are committed; and no point (zero) if three or more errors are committed on a particular string.

*Shapes Task.* This measure was originally developed by Kochanska *et al.* (1997), utilizing the Stroop paradigm (Rothbart, Deryberry & Posner, 1994) to measure inhibition. A modification of the Shapes Task was constructed to assess inhibitory control of children participating in this study. This consists of 24 8x11inch pictures representing large shapes (animals, fruits, household items). Each shape is cut out of paper covered with a design that depicts much smaller shapes. Geometric figures, numbers and letters present in the original version were excluded since most of the children have not yet learnt these concepts at the age of five years. In 12 trials (*consistent*), the small shapes are consistent with the large shape (e.g. a large moon made up of small moons) and in the other 12 trials (*inconsistent*) the shapes are inconsistent (e.g. a large cat made up of small bunnies or a large cow made up of small balls). The experimenter initially reviews all of the shapes with the child to ensure that he/she knows their names. The pictures are then presented one by one, and the child is to name as fast as possible the small shape in each picture. To prime the child’s bias to attend to the global shape rather than the smaller design, the inconsistent pictures are interspersed with the consistent ones. Scoring is done concurrently and scores were a pass for a correct response and a zero for an incorrect response. Scores on the 12 inconsistent trials were used for analysis.

*Tower of London.* This measure was adapted from Shallice’s version of Tower of London (Shallice, 1982) to test planning or problem solving. The task requires moving differently coloured balls across three equal sized pegs in order to duplicate a pre-specified target configuration. Three constraints apply: the child must not place more than the permitted number of balls on one peg, must not place the balls anywhere other than the peg, and only one ball is moved at a time. The TOL used in this sample comprised 10 trials altogether. Five of the trials were tower configurations in which a single tower was presented as the target, and the other five were of mixed configurations in which the design to be duplicated was spread over two or more pegs. The tower configurations were alternated with the mixed configuration trials. The first four trials required three moves, the next four trials four moves and the last two trials required five moves. Participant had to attempt all the trials although some children gave up before completion of the 10 trials. For a given trial, the child was asked whether they had finished constructing the configuration, and if they said ‘yes’, that trial was scored and they proceeded to the next trial. The score was the number of correct configurations out of the 10 completed trials hence a maximum possible score of 10. Children who failed to complete the 10 trials were not included in the analysis for this measure. Original scoring includes timing to the target configuration and the number of moves made. In this sample, however, timing and moves were excluded because pilot data showed that children did not differ greatly in terms of the time spent on the trials; therefore time was not a discriminating factor.

Pilot data for the extra measures showed near normal distribution of scores implying that the measures were sensitive to individual differences. Further, absence of floor or ceiling effects meant that these measures were of optimum difficulty. Final versions of the tasks were then compiled and equipment (scoring forms, pictures, TOL pegs and balls) was made. Descriptive statistics for performance on the extra measures using pilot data is shown on Table 7.

Table 7. Distribution of scores on the extra measures of executive function (pilot data) N=25

| Measure | Min | Max | Max possible | Mean | s.d |
| --- | --- | --- | --- | --- | --- |
| Counting Span | 3 | 8 | 8 | 5.32 | 1.54 |
| Running Memory | 9 | 18 | 20 | 13.28 | 2.19 |
| Shapes Task | 0 | 12 | 12 | 6.00 | 4.34 |
| Tower of London | 2 | 8 | 10 | 6.76 | 2.33 |
