# Supplementary material for: The role of the home environment in neurocognitive development of children living in extreme poverty and with frequent illnesses: a cross-sectional study
Source: Wellcome Open Res. 2018 Dec 3;3:152. [Version 1] doi: 10.12688/wellcomeopenres.14702.1 (PMC6338129; doi:10.12688/wellcomeopenres.14702.1)
Supplement: Peer review history

# Peer review: Referee response for version 1

**DOI:** 10.21956/wellcomeopenres.16011.r34580

**Published:** 2019-01-17

**Authors:** Robert H Bradley

**Affiliations:** 1 T. Denny Sanford School Social Family Dynamics, Arizona State University, Tempe, AZ, USA

As the authors note, there is a considerable literature showing that children’s development is influenced by their experiences at home.  However, a relatively small portion of that literature addresses the linkage between children’s health and neurocognitive development.  Moreover, most of the studies (as well as most of the measures used in studies) have been conducted in countries rated high on the Human Development Index (i.e., countries where the percentage of children living in deep poverty is relatively small).  Consequently, this study which examines relations between household SES, the home environment, neurocognitive development, and childhood illness in Uganda has much to recommend it.  The authors provide a reasonably comprehensive and up-to-date review of extant literature; and they provide reasonably convincing cases for the five statistical models used in the study.  That said, a good case can also be made for child health status as a potential moderator (not just mediator) of relations between SES, home environment, and neurocognitive development.  To some extent, the authors made such a case when they stated that the “effect” of the home environment on cognition was weaker for HIV infected children than controls ^1^. The value of considering a Model 6 (a multiple comparison form of SEM analysis) would also seem enhanced by considering the simple bivariate correlations shown in Table 5 and the fact that child health was not a significant mediator in the other models tested.  Another possibility would be a regression discontinuity analysis, doing a split on children’s health.

 The overall study design used was sound, particularly the effort to carefully examine the measures used to operationalize key constructs.  The approach used to adapt the HOME Inventory is particularly noteworthy, given the substantial differences in home life in Uganda versus the United States where HOME was developed.  The use of focus groups and local experts to determine the fit of existing indicators in HOME and the possible need for new indicators follows generally recommended practice and has been recommended by one of the developers of HOME ^2^.  The 68-item adapted version of HOME likely resulted in a much better estimate of relations between SES, child health, home environment, and child neurocognitive status than would have been obtained with the standard version of HOME.  That said, the overall statistical approach to evaluating HOME and SES was not optimal.  HOME is an index composed of formative or causal indicators.  It is not a scale composed of reflective indicators.  Consequently, the use of factor analysis (which requires reflective indicators) – as the researchers did -- and Cronbach’s alpha (a measure of unidimensionality) may not have produced clearly interpretable findings.  There have been advances in psychometric procedures for examining the structure and content of indices composed of formative indicators such as are contained in HOME ^3^ – the same is true of SES, which is also an index composed of causal indicators. 

 The use of SEM to examine the models corresponds to recommended practice for looking at mediational relations like those examined in the study.  The findings that emerged from the models tested are likely pretty robust; however, somewhat different findings may have emerged if additional psychometric analyses pertaining to HOME and SES were employed.  Moreover, there is concern about privileging the results of Model 5 primarily because all paths were significant.  It is, in fact, unlikely that all the impact of SES on child neurocognitive development is mediated through child health rather than through other relations via the home environment as well.  Again, an even stronger set of findings may have emerged had the researchers also included Model 6 as described earlier.  Although the general conclusions offered by the authors seem reasonable, somewhat different patterns of findings may have emerged had the study been longitudinal in design rather than cross-sectional.  Severe early health problems in some children may dominate the findings in this cross-sectional study, even though SES may impact neurocognitive development in children with less severe illness in other ways via the home environment over time.

 Despite some limitations in study design and statistical analysis, the broad findings from this study seem consistent with expectations and make even clearer the linkages likely present between SES, child health, home conditions and early neurocognitive development in children living in low HDI countries, like Uganda.  The findings offer an advance to knowledge in this area and should help guide a new generation of studies on these issues.  The results offer support for using a more holistic approach to understanding environment/development relations, consistent with ecological/developmental theory.

I have read this submission. I believe that I have an appropriate level of expertise to confirm that it is of an acceptable scientific standard.

## References

[1] Dobrova-KrolNAvan IJzendoornMHBakermans-KranenburgMJJufferF: Effects of perinatal HIV infection and early institutional rearing on physical and cognitive development of children in Ukraine. *Child Dev*.81(1) : 10.1111/j.1467-8624.2009.01392.x 237-51 10.1111/j.1467-8624.2009.01392.x 20331665

[2] BradleyRH: Constructing and Adapting Causal and Formative Measures of Family Settings: The HOME Inventory as Illustration. *J Fam Theory Rev*.2015;7(4) : 10.1111/jftr.12108 381-414 10.1111/jftr.12108 26997978PMC4795993

[3] BollenKABauldryS: Three Cs in measurement models: causal indicators, composite indicators, and covariates. *Psychol Methods*.2011;16(3) : 10.1037/a0024448 265-84 10.1037/a0024448 21767021PMC3889475

# Peer review: Referee response for version 1

**DOI:** 10.21956/wellcomeopenres.16011.r34374

**Published:** 2018-12-18

**Authors:** Michael J Boivin

**Affiliations:** 1 Departments of Psychiatry and Neurology & Ophthalmology, Michigan State University, East Lansing, MI, USA; 2 Department of Psychiatry, The University of Michigan, Ann Arbor, MI, USA

Summary and Conclusions of Study (provided by authors): This study investigated the relative influences of the long-identified connections between the home environment, child health and socio-economic status on a 5-year-old’s cognitive function in Ugandan children in very low-income settings. In line with the author's predictions, the data showed significant correlations among these three variables, and (with the exception of SES) these correlated with a wide range of cognitive tests. Using SEMs (AMOS), the authors tested a range of competing hypotheses about the nature of the interactions between these factors. While most of the models showed a good fit to the data, as assessed by RMSEA, only the one depicting a linear pathway from SES, through child health to HOME, showed significant links at all levels. The authors interpret these findings to mean that even where resources are limited and children are exposed to regular infections and diseases, their development can still be promoted by ensuring that they are provided with a stimulating home environment (especially modelling and academic stimulation).

 Critique Summary: The prior validation of the neuropsychological assessment battery in the present cultural context is a real strength in this study, and provides for valid cognitive performance outcomes. Likewise, the adaptation of the Caldwell HOME scale to evaluate quality of caregiving and developmental milieu for the study children is well done. Finally, the principal domains assessed for SES are appropriate and reasonable. The characterization of the health and medical history domain for the study children is less clear and not as fully detailed (e.g., anemia level, malaria history, parasite infections such as schistosomiasis and helminthes, treatment for malnutrition, prior hospitalizations). However, the SEM modeling is elegant and innovative, and makes a real contribution to this field of research. Furthermore, these findings and their interpretation are framed in an excellent review of the most relevant literature and theoretical models of the principal factors influencing early childhood development.

 The principal limitation to the study is that the great majority of children in this sample are HIV exposed (not clear if if their non-infected status was verified), born to mothers with HIV.  Therefore, this is a particularly at-risk population in terms of quality of care giving (with mother's health compromised by HIV), gestational and perinatal exposure to HIV (and likely combination ARVs), perhaps limited access to breast feeding because of maternal HIV status, impoverishment due to effects of HIV on the household, maternal depression and emotional well-being from HIV status, and nutritional status (in a subsistence agricultural setting where so much depends on the mother). 

 Given the above considerations for this study sample, it would be helpful to interpret how the principal SEM modeling findings might be different were the study children not HIV-exposed; for example, less significant to health factors as a direct linear mediator of SES effects in households not dramatically affected by HIV.

I have read this submission. I believe that I have an appropriate level of expertise to confirm that it is of an acceptable scientific standard.

## References

[1] BoivinMJBarlow-MoshaLChernoffMCLaughtonBZimmerBJoyceCBwakura-DangarembiziMRatswanaMAbrahamsNFairlieLGousHKamthunziPMcCarthyKFamiliar-LopezIJean-PhillippePCoetzeeJViolariACottonMCPalumboPEIMPAACT P1104s Study Team: Neuropsychological performance in African children with HIV enrolled in a multisite antiretroviral clinical trial. *AIDS*.2018;32(2) : 10.1097/QAD.0000000000001683 189-204 10.1097/QAD.0000000000001683 29112069PMC5736412

# Response

**Published:** 2018-12-18

**Authors:** Margaret Nampijja

**Affiliations:** MRC/UVRI and LSHTM Uganda Research Unit, Uganda

Thank you for your positive comments on our manuscript. We would like to highlight that the majority of the children (122,90.4%) were NOT HIV exposed.  As is indicated in the manuscript text and in Table 1, only 13 children (9.6%) were exposed to HIV infection  and of these only three children were found to be HIV positive. Hence the sample represents an HIV negative population and hence the results and discussions may be generalized to an HIV free population.
